# Physical activity phenotypes and mortality in older adults: a novel distributional data analysis of accelerometry in the NHANES

**DOI:** 10.1007/s40520-022-02260-3

**Published:** 2022-10-02

**Authors:** Marcos Matabuena, Paulo Félix, Ziad Akram Ali Hammouri, Jorge Mota, Borja del Pozo Cruz

**Affiliations:** 1 Centro Singular de Investigación en Tecnoloxías Intelixentes (CiTIUS), Universidad de Santiago de Compostela, Santiago de Compostela, Spain; 2 Research Center in Physical Activity, Health and Leisure (CIAFEL), Faculty of Sports, University of Porto (FADEUP), Porto, Portugal; 3 Laboratory for Integrative and Translational Research in Population Health (ITR), Porto, Portugal; 4 Centre for Active and Healthy Ageing, Department of Sports Science and Clinical Biomechanics, University of Southern Denmark, Odense, Denmark; 5 Faculty of Education, University of Cádiz, Cádiz, Spain; 6 Biomedical Research and Innovation Institute of Cádiz (INiBICA) Research Unit, Puerta del Mar University Hospital, University of Cádiz, Cádiz, Spain; 7 Epidemiology of Physical Activity and Fitness Across the Lifespan (EPAFit) Research Group, Faculty of Education, University of Seville, Seville, Spain

**Keywords:** Physical activity, Precision medicine, Accelerometry, Distributional representation, Longevity

## Abstract

Physical activity is deemed critical to successful ageing. Despite evidence and progress, there is still a need to determine more precisely the direction, magnitude, intensity, and volume of physical activity that should be performed on a daily basis to effectively promote the health of individuals. This study aimed to assess the clinical validity of new physical activity phenotypes derived from a novel distributional functional analysis of accelerometer data in older adults. A random sample of participants aged between 65 and 80 years with valid accelerometer data from the National Health and Nutrition Examination Survey (NHANES) 2011–2014 was used. Five major clinical phenotypes were identified, which provided a greater sensitivity for predicting 5-year mortality and survival outcomes than age alone, and our results confirm the importance of moderate-to-vigorous physical activity. The new clinical physical activity phenotypes are a promising tool for improving patient prognosis and for directing to more targeted intervention planning, according to the principles of precision medicine. The use of distributional representations shows clear advantages over more traditional metrics to explore the effects of the full spectrum of the physical activity continuum on human health.

**Supplementary Information:**

The online version contains supplementary material available at 10.1007/s40520-022-02260-3.

## Introduction

Physical activity is one of the most successful non-pharmacological interventions to promote the health of individuals, including the prevention and management of morbidity [1], and risk of early mortality [2]. Physical activity is also key to maintain an optimal functioning in older adults. Ultimately, engaging in recommended levels of physical activity is central to successful ageing [3].

Medical guidelines have traditionally promoted standard doses of moderate and vigorous intensity physical activity ranging from 150 to 300 min [4]. Recent advances in continuous monitoring technology (e.g., accelerometers) allow the recording, at a high level of resolution (e.g., second by second or minute by minute), of the amount and intensity of physical activity performed by an individual in a given period of time (e.g., a day or a week). Capitalizing on these advances, several epidemiological studies are yielding new findings with important clinical implications. For example, several studies have now revealed the role of light intensity physical activity in lowering the risk of early death and increasing the lifespan of the general population [2, 5–7]. Despite progress, there is still a need to determine more precisely the direction, magnitude, intensity, and volume of physical activity that should be performed daily to effectively promote the health of individuals [8–10].

Precision medicine is based on the idea of defining clinical phenotypes [11] or clusters of people who share a similar prognosis or response to treatments or other clinical events. These patient phenotypes are also helpful to define the different transitions of changes in individual health characteristics and classify the expected patient evolution more accurately. Unfortunately, to date, a few contributions that propose physical activity phenotypes using accelerometer data exist [12]. A better understanding of the health consequences of individual profiles of physical activity, using the full spectrum of accelerometry intensity across the day, would arguably help inform public health recommendations to promote the health of the population.

Benefiting from the abundant and unique information provided in the 2011–2014 National Health and Nutrition Examination Survey (NHANES) study, including the availability of high resolution accelerometry data, the current work aimed to define new physical activity phenotypes using an unsupervised clustering analysis in people aged 65–80 years. The secondary aim of this study was to ascertain the prospective associations of these phenotypes with 5-year survival probability and mortality. To achieve these aims, we capitalized on recently proposed distributional representations of accelerometry-based physical activity, which allows the quantification of time spent across the full spectrum of physical activity intensity without limiting to collapse the whole information into a few intensity intervals, as previously done using more traditional compositional metrics [13].

## Methods

### Sample

We used data from the NHANES waves 2011–2014. The NHANES aims at providing a broad range of descriptive health and nutrition statistics for civilian non-institutionalized population of the U.S. [14]. Data collection consists of an interview and an examination; the interview gathers person-level demographic, health, and nutrition information; the examination includes physical measurements, such as blood pressure, a dental examination, and the collection of blood and urine specimens for laboratory testing. Additionally, participants were asked to wear a physical activity monitor, starting on the day of their exam, and to keep wearing this device all day and night for seven full days (midnight to midnight) and remove it on the morning of the 9th day. The device used was the ActiGraph GT3X + (ActiGraph of Pensacola, FL).

A total of 2021 older adults aged 65–80 years (with physical activity monitoring available at least 10 h per day for 4 days) were included in the analysis. For the multivariate analysis, supported by additional biochemical, grip strength and comorbidities variables, 1064 participants were included due to missing data on covariates. In both cases, specific re-weight techniques on raw NHANES survey data were applied to properly handle the specific sampling mechanisms. The flow of participation in the current study is provided in the supplementary material.

### Sociodemographic and clinical data

Age (both as a categorical and continuous variable), race, gender, diagnosis of cancer or diabetes (as categorical variables), and blood pressure, combined grip strength measure, body mass index (BMI), and biochemical biomarkers, including cholesterol and triglycerides (as continuous variables), were considered in the analysis. Age was divided into three ranges (65–70, 70–75 and 75–80, respectively) for age-stratified analysis. Race variable was coded as 1 = Mexican American; 2 = Other Hispanic; 3 = Non-Hispanic white, 4 = Non-Hispanic black; 5 = Non-Hispanic Asian; and $6$ = Other Race, including multi-racial.

### Physical activity monitoring

Physical activity signals were pre-processed by staff from the National Center for Health Statistics (NCHS) to determine signal patterns that were unlikely to be a result of human movement. Then, acceleration measurements were summarized at the minute level using Monitor-Independent Movement Summary (MIMS) units, an open-source, device-independent universal summary metric [15].

Here, we adopt a novel representation of the resulting data that extends previous compositional metrics to a functional setting [16], aimed at overcoming their dependency on certain physical activity intensity thresholds. This approach also overcomes some previously known limitations of more traditional approaches.

Given a series of acceleration data $[({t}_{j},{x}_{j}){]}_{j=1}^{n}$ recorded in the interval $\left[0,T\right]$ over different monitoring periods, we propose to utilize a cumulative distribution function $F\left(x\right)$. Formally, consider a latent random process $Y\left(t\right)$, such that ${x}_{j}=Y\left({t}_{j}\right)$, $j=1,\dots ,n$, and define $F$ as

$$F\left(x\right)=\frac{1}{T}{\int }_{0}^{T}1\left(Y\left(t\right)\le x\right) dt,\text{for }x\ge 0.$$

We define the inactivity condition as ${P}_{inactive}=F\left(0\right)$, whilst ${F}_{active}\left(x\right)=F\left(x\right)-F\left(0\right)$ for $x>0$. Hence, $F\left(x\right)={P}_{inactive}+{\int }_{0}^{x}{f}_{active}\left(s\right)ds$, where ${f}_{active}=F{^{\prime}}_{active}\left(x\right)$. Thus, the continuous gait time is modeled through a density function, whilst inactivity time is modeled as a proportion. They can be easily computed from sample data in a two-step estimation procedure: first, the proportion of inactivity time is estimated as ${\widehat{P}}_{inactive}=\frac{{n}_{inactive}}{n}$, where ${n}_{inactive}={\sum }_{j=1}^{n}{1}_{\{{x}_{j}=0\}}$; second, the continuous physical activity profile is approached through a kernel density estimation

$${\widehat{f}}_{active}\left(x\right)=\left(1-{\widehat{P}}_{inactive}\right)\frac{1}{{n}_{active}}{\sum }_{j=1}^{n}{k}_{h}\left(x-{x}_{j}\right){1}_{\{{x}_{j}>0\}},$$

where ${k}_{h}\left(s\right)=\frac{1}{h}k\left(\frac{s}{h}\right)$ is a non-negative real-valued integrable function, $h>0$ is a smoothing parameter and ${n}_{active}={\sum }_{j=1}^{n}{1}_{\{{x}_{j}>0\}}$. In the present analysis, the Gaussian kernel was used for ${k}_{h}\left(s\right)$ and the smoothing parameter was selected through Silverman’s “rule of thumb” [17]. We finally used the quantile function estimator $\widehat{Q}\left(p\right)=inf\left\{x:p\le \widehat{F}\left(x\right)\right\}$, since they have proven to be particularly suitable for distributional modelling.

This new distributional representation allows us to measure the difference between physical activity profiles of different individuals by quantifying more comprehensively the amount of movement (i.e., acceleration, which resonates energy expenditure) over a given period and across the full spectrum of physical activity intensity.

### Mortality and survival

NHANES data can be linked to the National Death Index (NDI), enabling the study of the association between acceleration data, mortality status, and survival time. To this end, we accessed the 2015 Public-Use Linked Mortality Files [18], and included a binary variable indicating survival (or death) 5 years later, and the censored time to death.

### Statistical analysis

The primary goal was to identify a reduced set of clinically relevant phenotypes of physical activity supported by the new distributional representation and evaluate their impact on health. To this aim, we performed a clustering analysis using the kernel $k$-group algorithm [19]. To select the number of clusters, we used the well-established elbow rule [20]. According to this criterion, we estimated the within cluster sum of squares using the Gini mean difference for a different number of clusters, and we plotted the results. The number of clusters was then selected where there was a change in slope from steep to shallow (an elbow); in this case, *k* = 5.

We assessed the clinical relevance of these phenotypes to predict 5-year mortality and survival, and compared their clinical sensitivity and accuracy with age. We performed logistic and Cox regression on survey data. We then implemented the Kaplan–Meier estimator and included the phenotype as a categorical predictor. Odds ratios and hazard ratios, and graphical survival plots were used to quantify the prospective associations of these phenotypes on mortality and survival in the study sample.

Then, to remove the effect of potential confounding variables, we fitted again the logistic and Cox regression models and included also comorbidities, gender, race, cholesterol, and triglycerides as predictors in the models.

All statistical analyses were conducted using R software. Cluster analysis was performed using the Energy package, and survey analysis was performed using the Survey package.

## Results

### Physical activity phenotypes

Five clinical phenotypes were identified by means of a cluster analysis based on Euclidean energy distance. The optimal number of clusters was selected according to the rule-of-thumb [20].

**Fig. 1:**
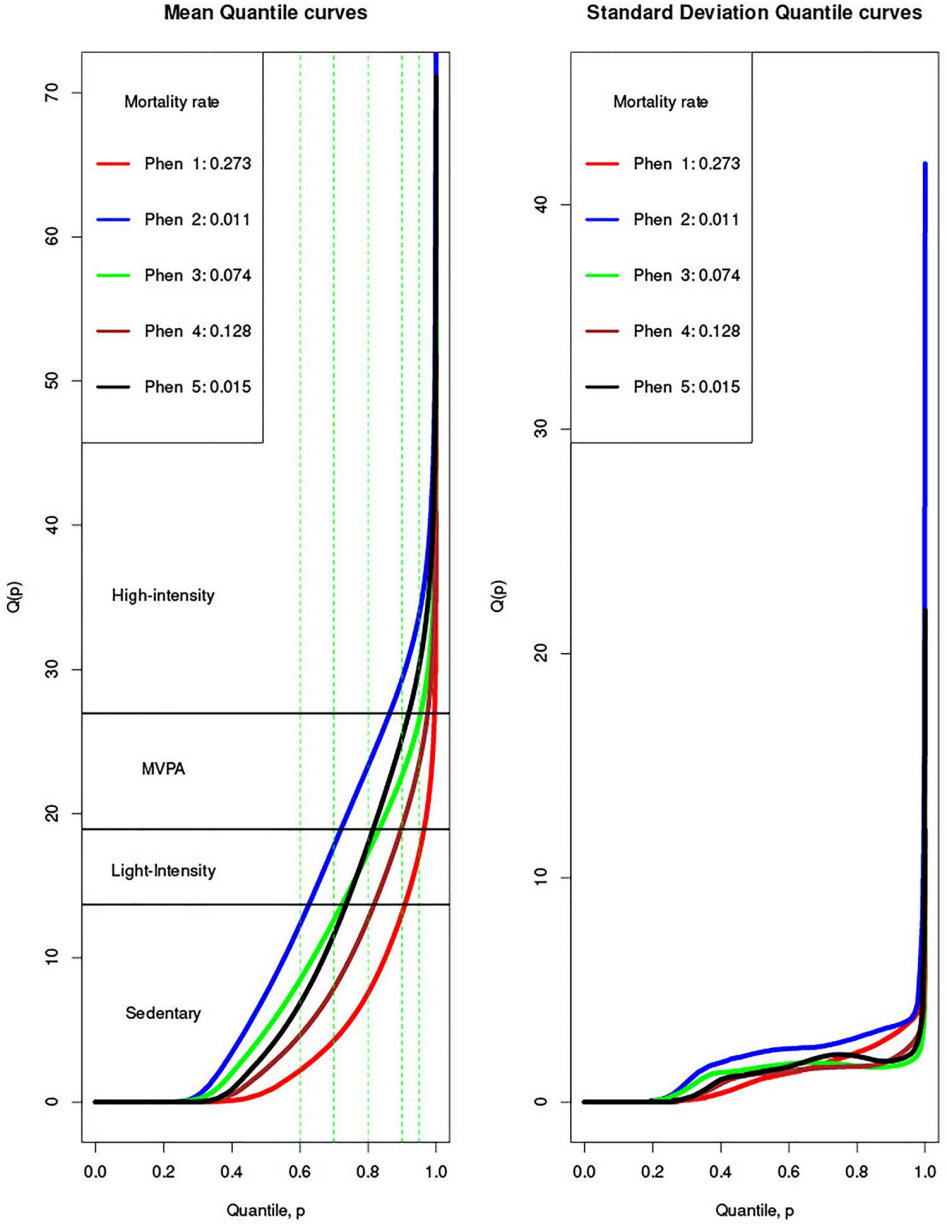
Mean and standard deviation of distributional representation for the five phenotypes together with their mortality rate

Figure 1 displays the mean quantile curves and the standard deviation quantile curves for the distributional representation of physical activity of each phenotype. The proportion of individuals who died after 5 years is also shown. We observed three phenotypes (Phenotypes 2, 3, and 5) with low mortality rate (less than 8%) and two phenotypes (Phenotypes 1 and 4) with a mortality rate of 27.3% and 12.8%, respectively.

The average distributional profiles of Phenotypes 1 and 4 showed a distinctive inactivity pattern: more than 80% of the time of participants in these two clusters is spent in sedentary behaviors (90% time vs. 80% time), with also important differences in the proportion of time spent in light and moderate-to-vigorous physical activity (MVPA) (5% vs. 10% and 2.9% vs. 6.5% respectively). Participants in Phenotypes 3 and 5 spent similar amount of time in sedentary (72% vs. 73%, respectively) and in light intensity (10% vs. 8%, respectively) activities, but Phenotype 3 had 5% more time in in MVPA. Finally, participants in the Phenotype 2, with the lowest mortality rate, only spent 62% percent of time sedentary, 10% in light intensity, 15% in MVPA, and 13% in higher intensities.

### Marginal survival analysis

**Fig. 2:**
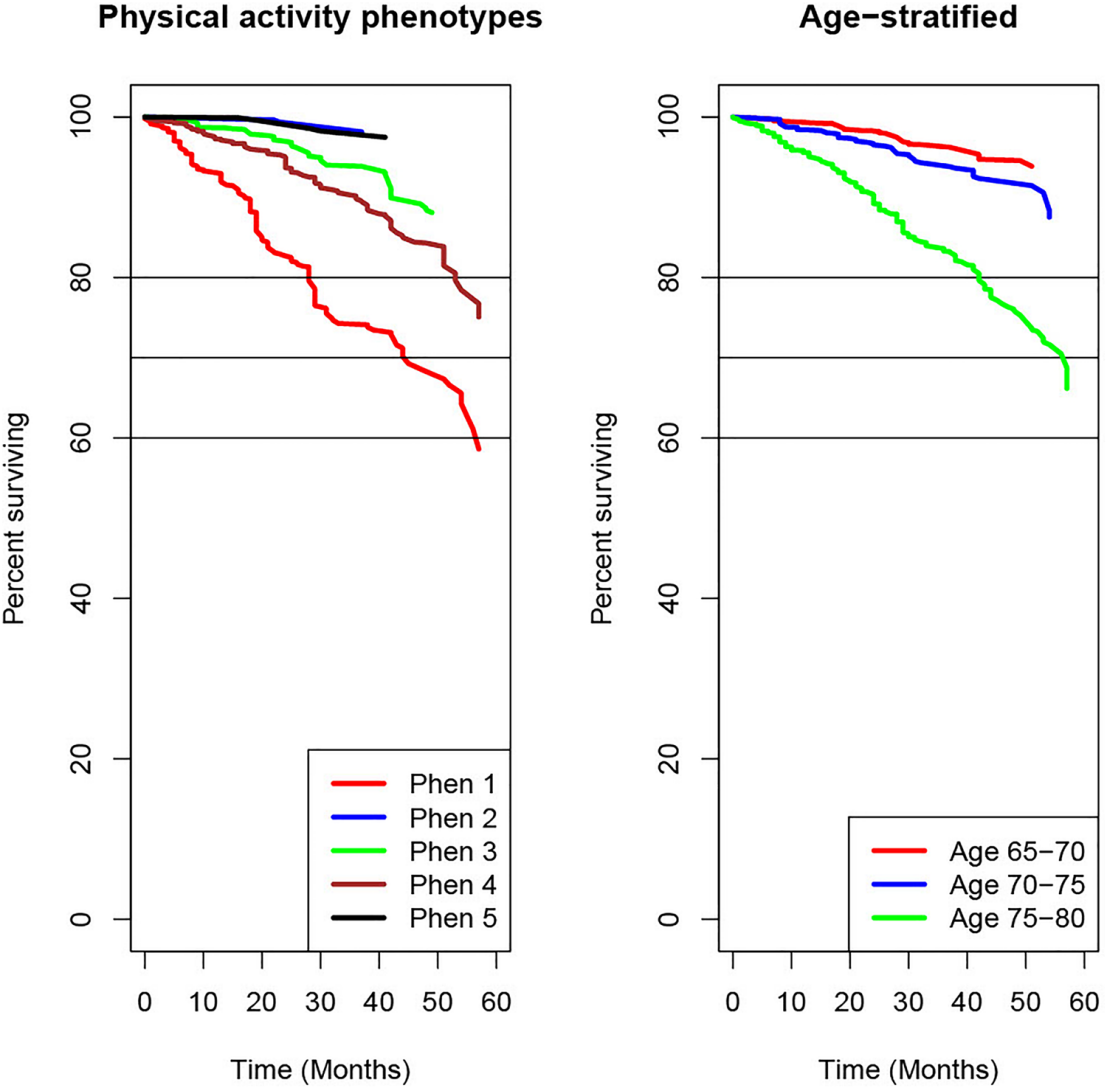
Kaplan–Meier curves for each phenotype and age group strata

Figure 2 displays a comparison of the survival curves for the different phenotypes and for the different age ranges. Participants in Phenotype 1 (the most inactive group) showed a lower survival compared with older individuals (75–80 years old). Table 1 shows the 5-year mortality and survival associated with each phenotype. Phenotypes 2–5 showed more than 90% less risk of mortality compared with Phenotype 1.

**Table 1:** Hazard ratios and Odds ratios (with 95% confidence intervals) of mortality outcomes associated with different physical activity phenotypes (reference: group 1—inactivity phenotype)

	Hazard ratio	2.5%	97.5%	Odds ratio	2.5%	97.5%
Phenotype 2	0.07	0.02	0.27	0.06	0.02	0.23
Phenotype 3	0.34	0.19	0.59	0.34	0.19	0.62
Phenotype 4	0.54	0.39	0.75	0.52	0.35	0.75
Phenotype 5	0.09	0.04	0.23	0.09	0.04	0.23
Age (years)	1.12	1.08	1.16	1.14	1.09	1.18
Gender	0.86	0.64	1.16	0.87	0.63	1.21

### Multivariate analysis

Population-based characteristics of the participants included in the multivariate analysis are shown in Table 2. Participants in Phenotype 1 were older on average than participants in the rest of phenotypes, and had a higher BMI, higher triglyceride level, and higher blood pressure. Phenotype 4, the second phenotype with more mortality rate, had a higher rate of diabetes and cancer, and the second higher BMI and age.

**Table 2:** Summary clinical characteristics of participants in each cluster

	Phenotype 1	Phenotype 2	Phenotype 3	Phenotype 4	Phenotype 5
Gender (men)	0*.*65	0*.*376	0*.*507	0*.*633	0*.*4664
High blood pressure	0*.*748	0*.*52	0*.*62	0*.*68	0*.*607
Cancer (yes)	0*.*273	0*.*224	0*.*258	0*.*286	0*.*162
Diabetes (yes)	0*.*531	0*.*5	0*.*484	0*.*591	0*.*492
No alcohol consume	0*.*064	0*.*039	0*.*095	0*.*243	0*.*307
Middle alcohol consume	0*.*07	0*.*097	0*.*175	0*.*408	0*.*661
High alcohol consume	0	0	0*.*008	0*.*016	0*.*026
Mexican American	0*.*042	0*.*096	0*.*054	0*.*042	0*.*068
Other Hispanic	0*.*021	0*.*136	0*.*072	0*.*062	0*.*11
Non-Hispanic white	0*.*669	0*.*488	0*.*552	0*.*615	0*.*618
Non-Hispanic black	0*.*197	0	0.303	0*.*216	0*.*126
Non-Hispanic Asian	0*.*049	0*.*144	0*.*014	0*.*039	0*.*068
Other race—including multi-racial	0*.*021	0*.*136	0*.*005	0*.*026	0*.*01
Age (years)	75*.*53 ± 4*.*87	69*.*38 ± 4*.*5	72*.*35 ± 5*.*21	73*.*1 ± 5*.*22	70*.*65 ± 4*.*76
BMI (Kg/m^2^)	29 ± 5*.*75	26 ± 4*.*85	28 ± 6*.*15	29 ± 5*.*69	28 ± 4*.*76
Combined grip strength (kg)	50*.*6 ± 18*.*9	55*.*7 ± 15*.*6	58*.*2 ± 19*.*1	59*.*5 ± 19*.*5	59*.*8 ± 18*.*4
Triglycerides (mg/dL)	1*.*84 ± 0*.*93	1*.*47 ± 0*.*92	1*.*59 ± 1*.*04	1*.*68 ± 1*.*06	1*.*74 ± 0*.*98
Cholesterol (mg/dL)	181*.*49 ± 43*.*88	191*.*46 ± 38*.*47	193*.*75 ± 39*.*69	179*.*07 ± 41*.*28	198*.*79 ± 43*.*84

In binary variables, we show the rate, and in continuous variables, we show the mean and standard deviation

Phenotype 1 (mortality rate of 27.3%) presented significant lower values of combined grip strength. However, Phenotype 4 (mortality rate of 12.8%) presented similar values of combined grip strength than the rest of physical activity phenotypes.

Table 3 shows the multivariate estimated coefficients (hazard and odds ratios) for mortality associated with physical activity phenotypes. Results remained consistent with univariate models presented in Table 1. Importantly, the confidence intervals for odds and hazard ratios do not cross 1, suggesting statistical significance.

**Table 3:** Results of logistics and Cox survey regression model in terms of odds ratio and hazard ratio

	Hazard ratio	2*.*5%	97*.*5%	Odds ratio	2*.*5%	97*.*5%
Phenotype 2	0*.*12	0*.*02	0*.*66	0*.*10	0*.*02	0*.*62
Phenotype 3	0*.*29	0*.*11	0*.*75	0*.*30	0*.*11	0*.*80
Phenotype 4	0*.*55	0*.*31	0*.*98	0*.*49	0*.*25	0*.*98
Phenotype 5	0*.*07	0*.*01	0*.*61	0*.*07	0*.*01	0*.*62
Age	1*.*10	1*.*04	1*.*17	1*.*12	1*.*05	1*.*19
Gender (woman)	0*.*92	0*.*57	1*.*47	0*.*99	0*.*61	1*.*63
Other Hispanic	0*.*81	0*.*14	4*.*49	0*.*69	0*.*11	4*.*38
Non-Hispanic white	0*.*79	0*.*35	1*.*80	0*.*60	0*.*25	1*.*43
Non-Hispanic black	0*.*43	0*.*15	1*.*21	0*.*35	0*.*12	1.05
Non-Hispanic Asian	1*.*13	0*.*31	4*.*12	1*.*17	0*.*27	5*.*06
Other race, including multi-racial	1*.*21	0*.*35	4*.*14	1*.*18	0*.*29	4*.*76
Blood pressure hight	0*.*84	0*.*40	1*.*80	0*.*77	0*.*35	1*.*71
BMI	0*.*99	0*.*93	1*.*06	0*.*99	0*.*93	1*.*06
Middle Alcohol	0*.*55	0*.*33	0*.*92	0*.*60	0*.*33	1*.*10
High alcohol	0*.*97	0*.*12	8*.*17	1*.*68	0*.*15	18*.*64
Cancer (no)	0*.*84	0*.*48	1*.*47	0*.*95	0*.*51	1*.*75
Diabetes (no)	0*.*89	0*.*59	1*.*35	0*.*91	0*.*58	1*.*44
Triglycerides	0*.*80	0*.*54	1*.*19	0*.*79	0*.*54	1*.*17
Cholesterol	1*.*00	0*.*99	1*.*00	1*.*00	0*.*99	1*.*00

The interval confidence of coefficients is estimated with a level of 95%. Reference Group 1—inactivity phenotype

## Discussion

This paper reveals new physical activity phenotypes for the U.S. older population using novel distributional representations of accelerometer-derived physical activity. The new clinical phenotypes yield a higher clinical sensitivity for predicting 5-year mortality and survival outcomes than age alone. Our results show that the most inactive physical activity phenotype has a much lower survival probability than the oldest participants in our sample.

Our findings reinforce the idea that information related to physical activity is a key non-pharmacological biomarker of functional decline status and general health [21, 22]. Previous studies [7] have shown the greater clinical sensitivity of physical activity to predict 5-year mortality with the NHANES data 2003–2006 (compared to age), although such level of performance was not observed in the UK-Biobank study [5]. This discrepancy is likely due to the limitation of UK-Biobank study design and the selection bias. Our results were confirmed in multivariate analyses adjusting for potential confounders, such as age, race, sex, comorbidities, or biochemical variables, such as cholesterol or triglycerides. We also derived specific weights for the sample included in the analysis, thereby reinforcing the generalizability of our results.

The introduction of new clinical phenotypes with the novel distributional representations allowed us to assess the amount of movement along each intensity recorded by the accelerometer monitor, unlike other existing compositional metrics used in the literature [14]. The summary functional curves (mean and variance) derived from the cluster analysis done in our study show differentiated patterns of physical activity, with remarkable differences across the intensity spectrum from inactivity; and highlight the need to monitor and quantify physical activity more precisely, also to detect the impact on health of intensities often hidden in previous, threshold-based monitoring of physical activity. The phenotypes generated in this study may serve as a formal framework to assess activity changes, for example, with an intervention. In this sense, it is worth mentioning that a reduction in mortality risk between two of the phenotypes might only be due to an increase in the MVPA duration. In addition, the generated phenotypes could be used as a prognosis and monitoring tool. Our work adds to the (yet scarce) number of works that have explored the idea of physical activity phenotypes as a health monitoring tool [12].

A recent review indicated that there may not exist solid evidence of the benefits of physical activity in patient prognosis in some diseases, such as cardiovascular problems [2]. However, it is remarkable to note the sizeable individual response of patients to physical activity and that patients with standardized training programs improve fitness and not necessarily maximal oxygen uptake [23–25]. Several investigations have shown the relationship between maximal oxygen uptake and the prognosis of these patients and their survival and risk of mortality [23]. Thus, monitoring patient profiles at a high level of resolution is essential to ensure the optimal prescription of physical activity. Indeed, some recent works showed the protective role of light intensity activity for longevity [6, 26]. In addition, the health impact of the optimal intensity–volume coupling is the result of a complex process influenced by many factors, such as genetic and environment, which must be considered in exercise prescription [8, 9]. In this regard, the new patient stratification methods may provide a framework for analyzing these factors and guiding training prescription.

The main strength of this study is that the data used are a random sample from a complex survey design, unlike a significant fraction of physical activity studies that use observational data. Thanks to the NHANES survey design, we can obtain more general conclusions about the impact of physical activity on health profiles of the U.S. population. The sample size is another strength, although other cohorts, such as the U.K-Biobank, have a more significant number of participants; yet its experimental design has inherent limitations.

Distributional representations provide further advantages in statistical modelling, since they intrinsically capture the information represented by compositional metrics [16, 27, 28] and lead to more refined physical activity profiles which expand along the continuous spectrum of intensity. In addition, the new and more sophisticated pre-processing of accelerometer data leads to greater sensitivity [15], especially for detecting differences in light- and high-intensity physical activity.

An inherent limitation of this study is the non-incorporation of potential confounders, such as genetic variables, but this is present also in other observational studies. In addition, with a more extensive physical activity monitoring period, we could have drawn more reliable conclusions about the impact of individual physical activity patterns on health. However, in this paper, we analyzed older individuals with lower functional capacity, and this could limit the impact of intraday variability in physical activity patterns (i.e., our population may show more consistent patterns of physical activity than younger and fitter populations). Similarly, the non-inclusion of the temporal component of distribution representations is another added problem that may lead to new findings of the role of physical activity on health. For example, recent studies have shown the effects of the chronobiology differences in physical activity on health [29].

In summary, this study provides new phenotypes in the ageing U.S. population and shows their clinical utility to predict the mortality and survival outcomes in the study sample. Following the principles of precision medicine [30], and according to the phenotypes obtained, differences in light and high-intensity physical activity are relevant for health. The use of distributional representations could be advantageous over more traditional threshold-based analytical approaches to explore the effects of physical activity on human health.

## Supplementary Information

Below is the link to the electronic supplementary material.

Supplementary file 1: **Figure** Flow of participation in the present study

## Data Availability

The data used here can be freely downloaded from the NHANES website, and Disease Control and Prevention has conducted the study under U.S. law. In case of a reasonable request, the author provides the processed data and scripts. All data are publicly available from CDC NHANES Database. https://wwwn.cdc.gov/nchs/nhanes/Default.aspx.
