# Supplementary figures and images for: Physical activity phenotypes and mortality in older adults: a novel distributional data analysis of accelerometry in the NHANES
Source: Aging Clin Exp Res. 2022 Oct 2;34(12):3107–14. doi: 10.1007/s40520-022-02260-3 (PMC9719452; doi:10.1007/s40520-022-02260-3)

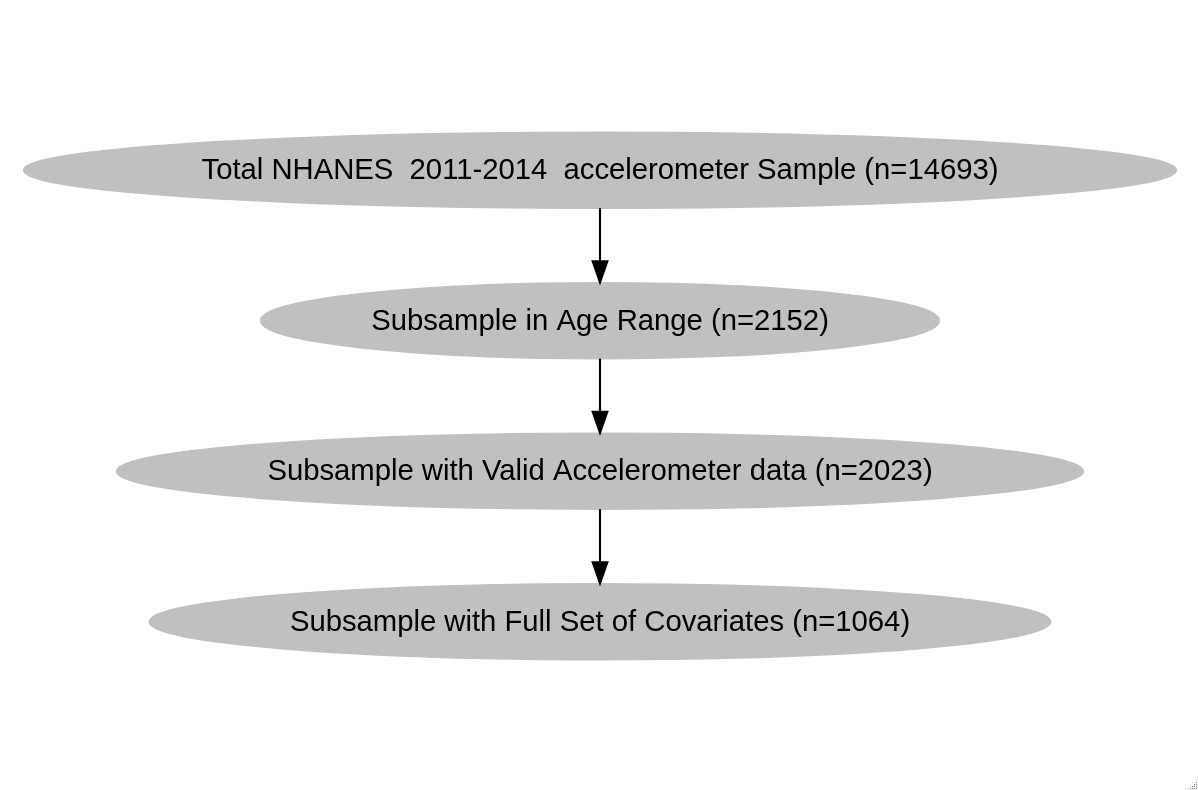

Supplement: Supplementary file 1 — Figure Flow of participation in the present study [file 40520_2022_2260_MOESM1_ESM.png]
